# Evaluation of an ultra-portable pocket-sized device for running Loop mediated isothermal amplification (LAMP) assays for rapid detection of sweetpotato viruses

**DOI:** 10.12688/gatesopenres.16369.1

**Published:** 2025-10-24

**Authors:** Segundo Fuentes, Kwame Ogero, Ana Perez, Jan Frederick Kreuze

**Affiliations:** 1 International Potato Center, Lima, Peru; 2 International Potato Center, Mwanza, Tanzania

**Keywords:** virus detection, pocket LAMP, portable device, Genie, SPFMV, SPCSV, SPLCV

## Abstract

The sweetpotato ( *Ipomoea batatas*) is an important food crop in the tropical and subtropical regions of the world, but its yield and quality are heavily affected by viral diseases. Timely and precise detection of virus infections is essential for effective monitoring of seed health and disease management. We evaluated the feasibility of using a compact, ultra-portable LAMP-based diagnostic device—initially designed for human health applications—for detecting key sweetpotato viruses (SPCSV, SPFMV, and SPLCV). Field and greenhouse samples were tested, showing 100% agreement in virus detection with a larger commonly used LAMP device. Sensitivity tests confirmed consistent performance, and the use of portable power banks enabled reliable on-site use. The statistical analysis indicated high accuracy and strong correlation in time-to-positive values between methods (r > 0.89, p < 0.01). Furthermore, cost analysis demonstrated that the pocket LAMP device setup significantly reduced per-test costs—by approximately 40%—while maintaining diagnostics reliability. These findings support the potential of this tool on plant virus detection in locations with limited resources.

## Introduction

Sweetpotato ( *Ipomoea batatas* (L.) Lam) is an important cash and food crop around the world with a particular significance in tropical and semi-tropical climate ( O’Brien, 1972; Lebot, 2010). Nevertheless, its production is challenged by many factors including viral diseases that compromise crop yield and quality ( Untiveros et al., 2007; He et al., 2023; Gutiérrez et al., 2003; Kreuze et al., 2024). More than 35 viruses infecting sweetpotato have been reported worldwide ( Clark et al., 2012; Kreuze et al., 2021), but a few, such as Sweet potato chlorotic stunt virus (SPCSV), Sweet potato feathery mottle virus (SPFMV), and Sweet potato leaf curl virus (SPLCV), are particularly prevalent and damaging globally. These viruses not only reduce crop yields on their own but also interact with each other, making disease symptoms much more severe and leading to even greater economic losses ( Clark et al., 2012; Cuellar et al., 2015; Kreuze et al., 2024; Ogero and van der Vlugt, 2023; Wanjala et al., 2020).

The proper and strict virus detection which is carried out in the seed certification programs and breeding efforts is essential for managing these diseases. A number of diagnostic techniques have been developed including biological indexing where the indicator plants are used, serological assays such as enzyme-linked immunosorbent assay (ELISA), and molecular tests such as reverse transcription polymerase chain reaction (RT-PCR), real-time PCR (qPCR), loop-mediated isothermal amplification (LAMP), microarrays, and small RNA sequencing and assembly (sRSA) ( Kreuze, Cuellar, and Low, 2021; Kreuze et al., 2024; Kanapiya et al., 2024; Loebenstein, 2015; Mbewe et al., 2021; Tibiri et al., 2020). While these laboratory-based methods offer high sensitivity and specificity, they need specialized facilities, trained personnel, and extended processing times, often ranging from several hours to days or weeks ( Clark et al., 2012; Kanapiya et al., 2024; Devi et al., 2024). These limitations indicate the need for portable, low-cost, and quick-deployable virus diagnostic methods that can be utilized in the field.

LAMP is gaining popularity as a reliable alternative to other methods of plant virus diagnosis because it is fast (results in 20–60 minutes), simple, and requires minimal equipment ( Notomi et al., 2000; Wanjala et al., 2024). LAMP assays have been effectively tested for on-site virus detection in various crops, including sweetpotato ( Kreuze et al., 2024; Wanjala et al., 2024). Their reliability makes them especially valuable for ensuring the quality of early-generation seeds ( Wanjala et al., 2024).

An inexpensive, portable pocket LAMP device — ‘DoctorVida’ originally designed to detect SARS-CoV-2 ( Doria et al., 2022) developed by STAB VIDA (Portugal) — was included in preliminary comparative tests conducted in 2022. This smartphone-operated device was evaluated alongside the commonly used case-portable ‘Genie III’ device ( Quoc et al., 2018; Lee et al., 2020; Wanjala et al., 2024; Rako et al., 2023; Wharton et al., 2025), using the same reagents and SPCSV-infected samples. The result showed that the pocket LAMP device can be effectively adopted and adapted for sweetpotato virus detection.

Our results demonstrate that the pocket LAMP device reliably detects major sweetpotato viruses (SPCSV, SPFMV, and SPLCV) using LAMP technology. Its performance is equivalent to that of commonly used larger case-portable devices, offering a convenient and effective alternative for virus detection in sweetpotato, contributing to improved disease management.

## Materials and methods

### Samples collection

In August 2023, three sweetpotato fields (1.5–2.5-month-old plants) were surveyed in Huaral, Lima, Peru. From each field, 40 plants with and without symptoms (vein clearing, mosaic, leaf deformation, chlorosis, etc.) were chosen as samples. Symptoms were documented with photographs, and from symptomatic plants, three leaves of varying symptoms severity were sampled to represent different viral loads present in the tissues. For asymptomatic plants, one leaf each from the top, middle, and bottom was sampled.

Leaves were placed between filter paper, desiccated with orange silica gel and stored at room temperature. Out of the 120 field-collected samples, 88 well-preserved dried leaves samples were selected for LAMP assays. Initial testing detected only SPCSV and SPFMV. To further investigate, an additional 25 fresh samples were collected from screenhouse-grown plants (germplasm collection) exhibiting leaf curl symptoms at CIP-Lima. These samples were specifically evaluated for SPLCV by LAMP assays.

### Sample processing

From the sample (composed of three desiccated leaves) a piece representing 1cm-disc in diameter was taken from each leaf and placed in a polyethylene sample bag. Then, 1 ml of the alkaline PEG buffer ( Chomczynski and Rymaszewski, 2006) was added into the polyethylene sample bag to macerate the tissue. Then, sap extraction was diluted 1:10 (v/v) in nuclease-free water (NFW). Fresh tissue was used from samples collected in the screenhouse.

### LAMP assays

**Figure 1.**
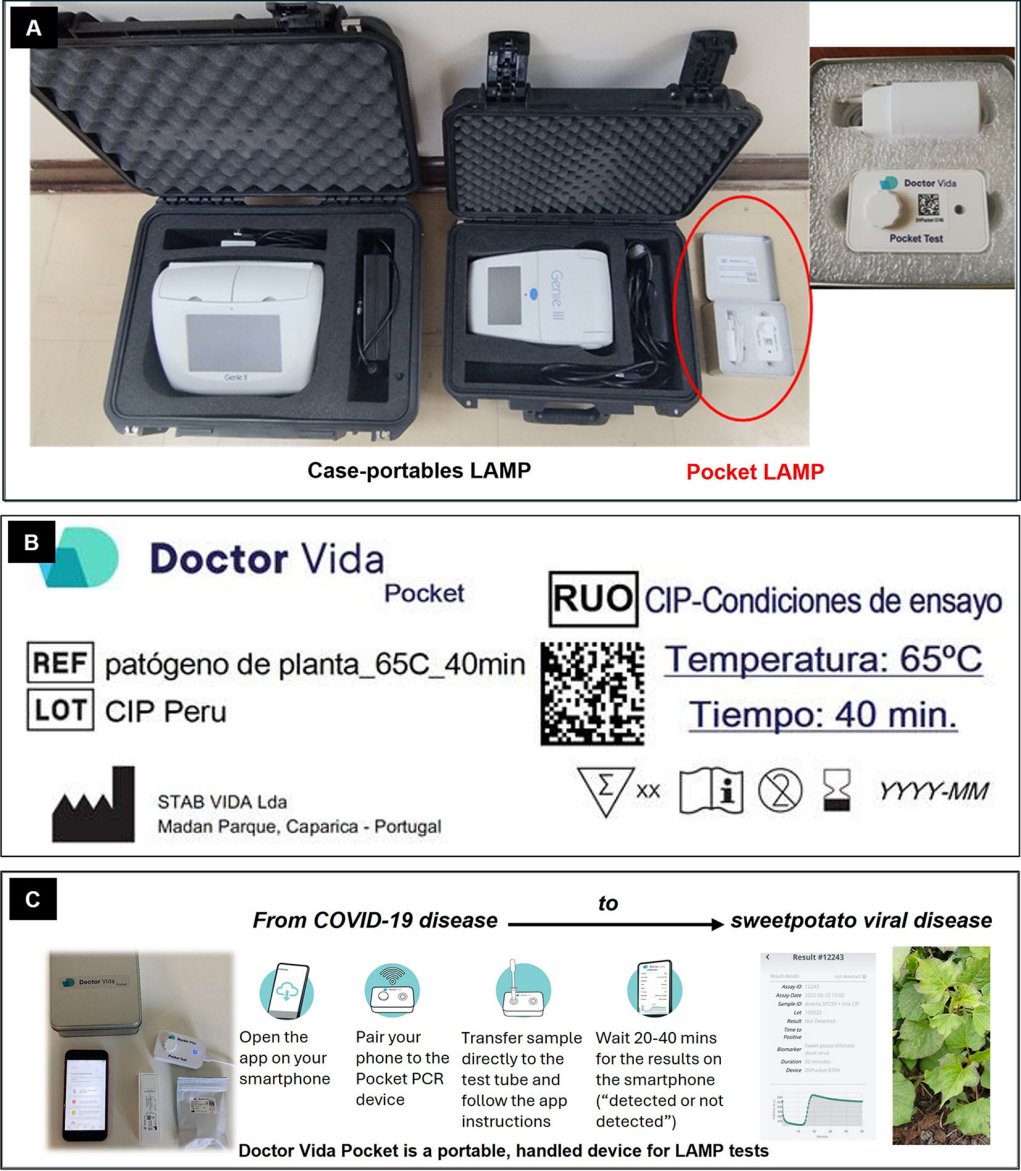
Overview of pocket LAMP diagnostic device. **(A)** Size comparison with case-portable LAMP devices. **(B)** LAMP test configured at 65 °C for 40 minutes, using a specific master mix and fluorescence measurement with a blue filter. **(C)** Workflow of the testing process (adapted from STAB VIDA).

The pocket LAMP device was set up in collaboration with the supplier to enable detection of sweetpotato virus, with protocol providing via QR code and installing the corresponding firmware on the devices ( Figure 1). LAMP assays followed Wanjala et al. (2024), using filter tips to prevent contamination. Master mix reactions (Supplementary Table S1, Fuentes et al., 2025) were prepared using master mix ISO-DR004-RT or ISO-001 (OptiGene) with six primers per virus (SPFMV, SPCSV, SPLCV) and cytochrome oxidase (COX) as an internal control (Supplementary Table S2, Fuentes et al., 2025). The reaction mix (master mix and diluted sample) was combined and homogenized in a single Eppendorf tube before dispensing in OptiGene tubes for the case-portable ‘Genie III’ device (25 μl) and in 0.2 ml PCR tubes (50 μl; Axygen, flat cap) for the pocket LAMP device. In the latter case, 50 μl hexadecane (Sigma-Aldrich) was added on top to prevent evaporation.

**Figure 2.**
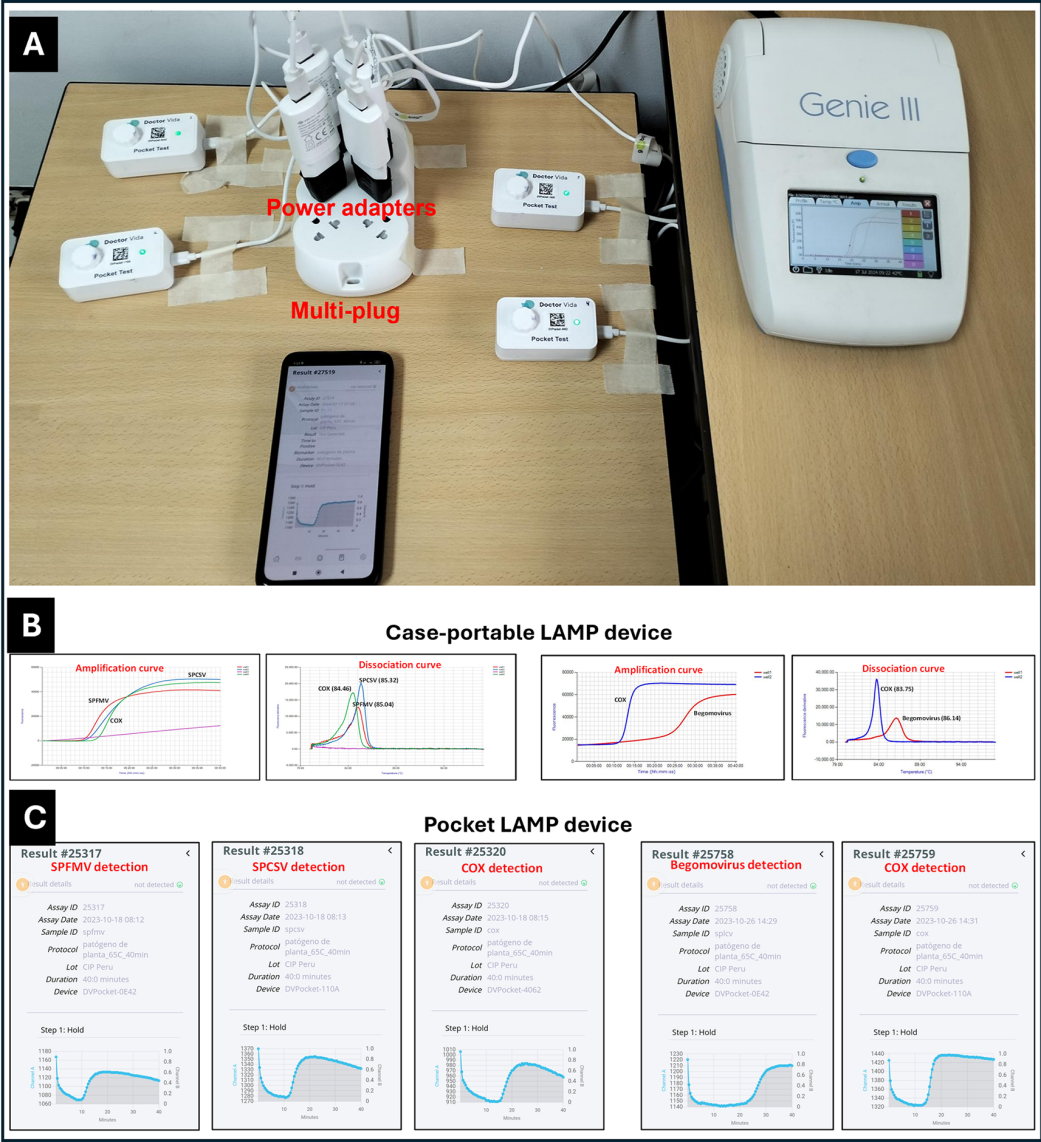
Comparative performance of two LAMP devices for sweetpotato virus detection. **(A)** Side-by-side testing of SPFMV, SPCSV, SPLCV, and COX control on the devices plugged into electrical power. **(B)** Amplification curves from the case-portable ‘Genie III’ device with 25 μl of reaction mix in Genie tubes. **(C)** Amplification curves from the pocket LAMP device with 50 μl of reaction mix layered with 50 μl of hexadecane to prevent evaporation. Positive reactions are visualized as amplification curves.

In addition, for ensuring consistency, the assays were initiated simultaneously on both devices ( Figure 2). LAMP assays were performed at 65 ^o^C for 40 min, followed by a melting step from 75 to 95 ^o^C (for Genie III only). Each device was operated per manufacturer’s instructions ( Genie III, DoctorVida).

For sensitivity assays, 10 dried samples identified as infected with SPFMV and SPCSV were selected from field samples tested previously by LAMP assays. A 100 mg tissue sample (from three desiccated leaves) was macerated in 1 ml alkaline PEG buffer. The sap extract was diluted 1:10 (v/v) in NFW, which was considered as 1:1, then serially diluted 20-fold (1:20, 1:400, 1:8,000) in 0.1X alkaline PEG buffer.

**Figure 3.**
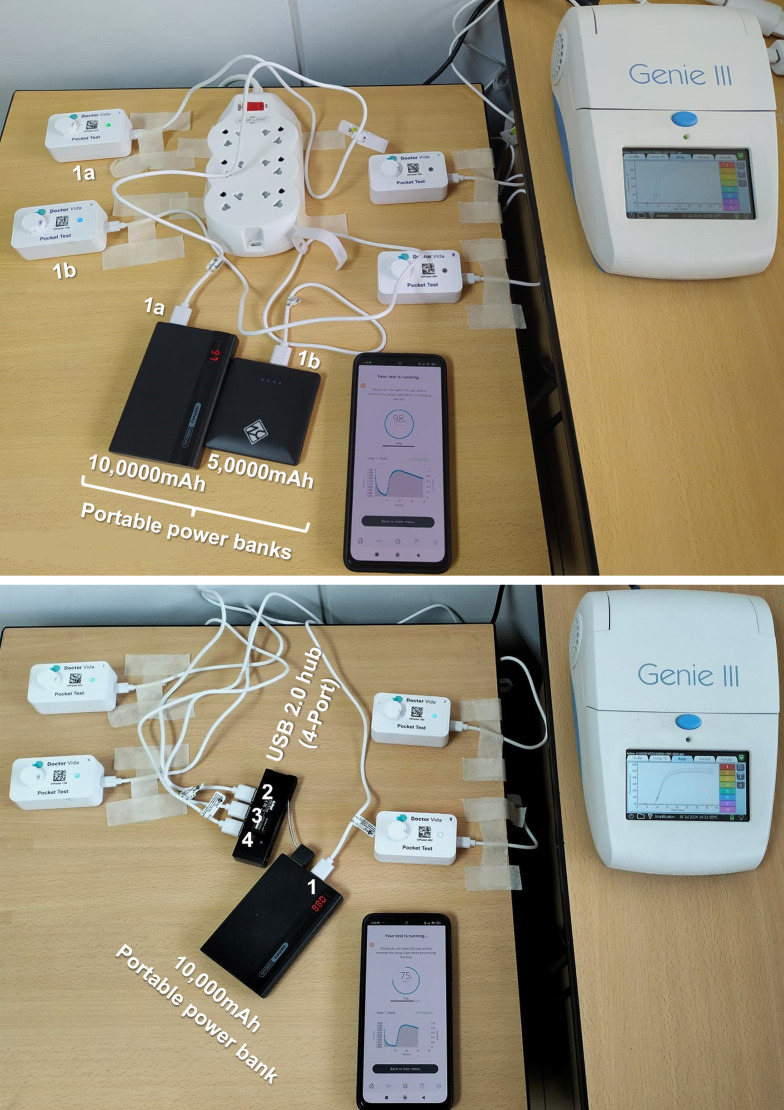
Performing simultaneous LAMP tests on two diagnostic devices for comparing detection results. **(Top)** A single pocket LAMP device (1a and 1b) powered by a portable power bank (1a and 1b, respectively). Note the green light emission of devices on the left side. **(Bottom)** Four pocket LAMP devices connected to a single power bank: one directly and three via a USB 2.0 (4-port) hub.

For assays with portable power supply, the pocket LAMP devices were powered by 5,000mAh (Refuel Max) and 10,000mAh (REMAX) cell phone power banks. Compatibility was confirmed by matching the device adapter’s output (5V, 2.0A) with that of each power bank ( Figure 3). As in prior tests, the same samples and master mix reactions were used to ensure consistency.

### Correlation analysis

A comparative analysis was conducted between the two diagnostic devices, the case-portable ‘Genie III’ and the DoctorVida pocket LAMP device. Results were recorded as binary outcomes, with “0” for negative reaction and “1” for positive reaction. Time to positive (TTP) was obtained directly from Genie III, while for the pocket LAMP device, TTP was manually extracted from graphs stored on the supplier’s server.

To assess agreement between the two devices, positive and negative results were counted and compared, and correlation analysis along with Deming regression on TTP values was performed ( Kreuze et al., 2024). Deming regression fitted using the “mcr” R package (v3.3.1; Potapov et al., 2023), accounted for measurement error in both methods. The estimated intercept and slope suggested systematic and proportional differences. A bootstrap-based method was used to calculate 95% confidence intervals for the regression parameters, as well as the confidence region for the regression line, providing quantitative assessment of uncertainty and a mean to assess the suitability of a linear calibration curve.

### Cost-effectiveness analysis

A cost-effectiveness analysis was performed to assess the expenses associated with using the pocket LAMP device as compared to Genie III (including its carry case) as the benchmark. The case-portable ‘Genie III’ device can perform eight LAMP tests simultaneously, whereas each pocket LAMP device processes one test at a time. Therefore, running eight tests simultaneously would require eight pocket LAMP devices and two smartphones (each phone can control up to four devices). Costs were estimated based on local pricing in Tanzania.

The set-up included additional necessary accessories such as two multi-4-plug adapters with eight power adapters, internet service for 20 weeks (cost depending on the number of samples tested, under Tanzania context), two 10,000mAh portable power banks for on-site assays, and two Pluggable USB 2.0 4-Port Hubs ( Figure 2 and Figure 3).

The cost analysis also factored in consumables essential to each assay, including device-specific 0.2 ml tubes, ISO-DR004-RT master mix, and hexadecane. The variable-cost consumables analysis was based on 2,000 tests, sufficient to test 500 samples, with four tests per sample (three viruses plus COX).

## Results

### Virus detection

LAMP assays performed using the case-portable device, which combined amplification curves with dissociation/melting curves, were used as a reference to confirm specific reactions with the target viruses and the COX control. Supplementary Table S3 ( Fuentes et al., 2025) summarizes the LAMP results obtained from both the pocket LAMP and case-portable device, highlighting the correspondence between them. All tested samples (113) showed positive reactions for COX, confirming the quality of the sap extraction and the absence of inhibition in the LAMP assays.

Virus detection results obtained using the pocket LAMP device were fully consistent with those from the case-portable, showing 100% concordance. Specifically, 9 samples tested positive for SPFMV, 68 samples for SPFMV+SPCSV, 17 for SPLCV, and 19 samples were negative for any of the targeted viruses (Supplementary Table S3, Fuentes et al., 2025).

Fresh leaves from 17 screenhouse-maintained samples tested positive in the LAMP assays ( Figure 2 and Supplementary Table S3, Fuentes et al., 2025). However, after drying, these samples tested negative for SPLCV.

### Sensitivity assays

**Figure 4.**
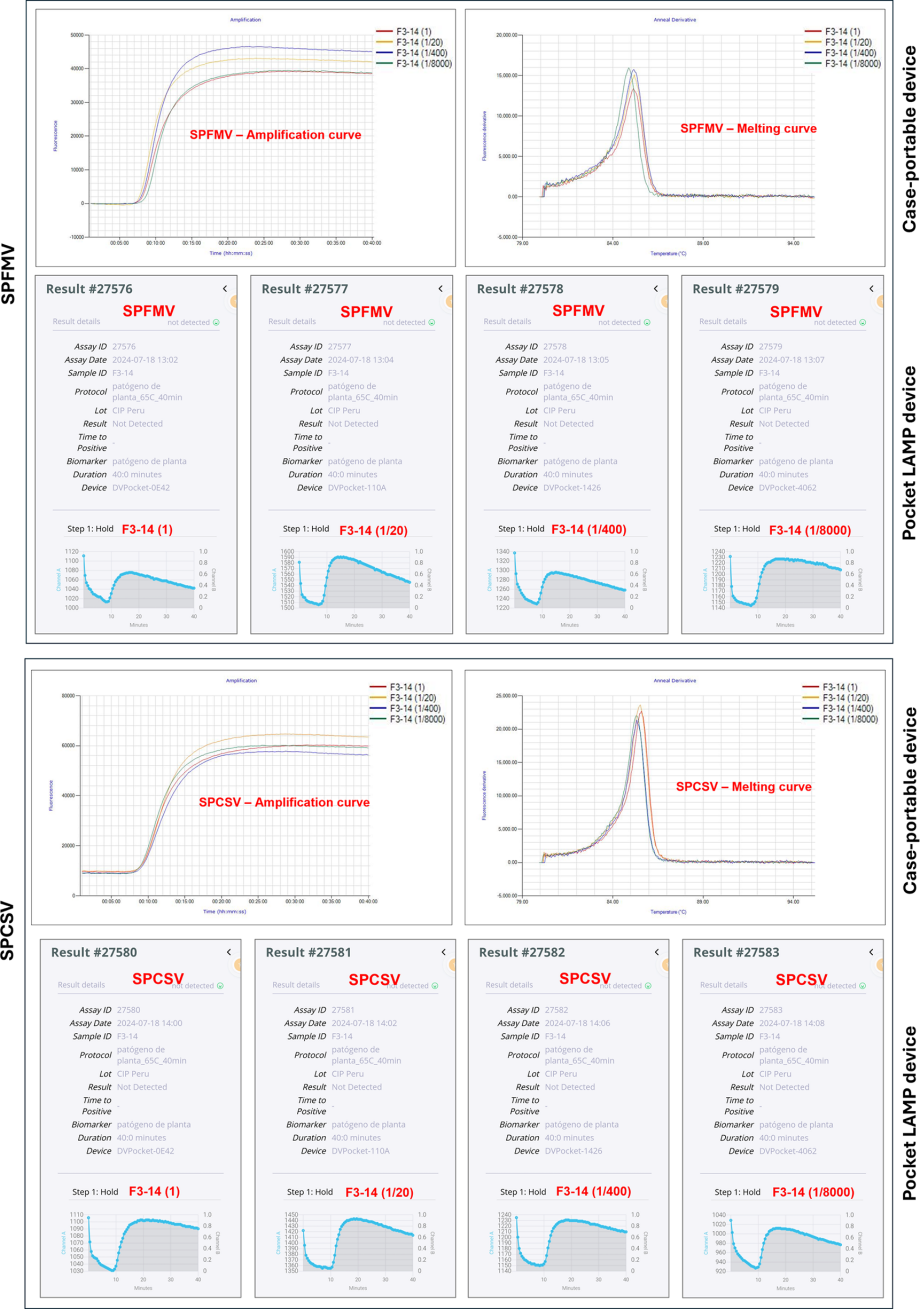
Comparison of SPFMV and SPCSV detection by LAMP tests using two detection devices. Four pocket LAMP devices were powered by a REMAX 10,000 mAh portable phone power bank. LAMP tests were conducted simultaneously with sample F3-14 to compare results.

All tested samples showed positive reactions for SPFMV and SPCSV, as expected. The detection results from the pocket LAMP device were fully consistent with those from the case-portable device, showing 100% concordance in virus detection. The time to positivity (TTP) was similar between both devices. Except for one sample (F1-11), no significant differences were observed in the TTPs across the various sap dilutions ( Figure 4). These results suggest that any LAMP assays from both devices can be conducted appropriately, providing reproducible results. Supplementary Table S4 ( Fuentes et al., 2025) presents the LAMP test results obtained by both devices.

Samples selected for SPLCV detection were negative but successfully amplified the internal control COX, confirming the quality of sap extraction and the absence of inhibition in the LAMP tests. Additionally, the SPLCV positive control (using either fresh leaves or nucleic acid extraction) produced the expected amplification results.

### Performance using portable power banks

The pocket LAMP device functioned effectively when powered by portable cell phone power banks, performing similarly to when connected to an electrical outlet. One to four devices operated well with a single power bank ( Figure 4).

A LAMP run (40 minutes for amplification and 7 minutes for melting) with four devices connected to a REMAX 1000mAh power bank consumed approximately 20% of its charge, leaving enough power for at least three additional assays. This capability supports on-site LAMP assays for detecting sweetpotato viruses, provided internet access is available for saving and uploading results to the cloud.

### Correlation analysis

**Figure 5.**
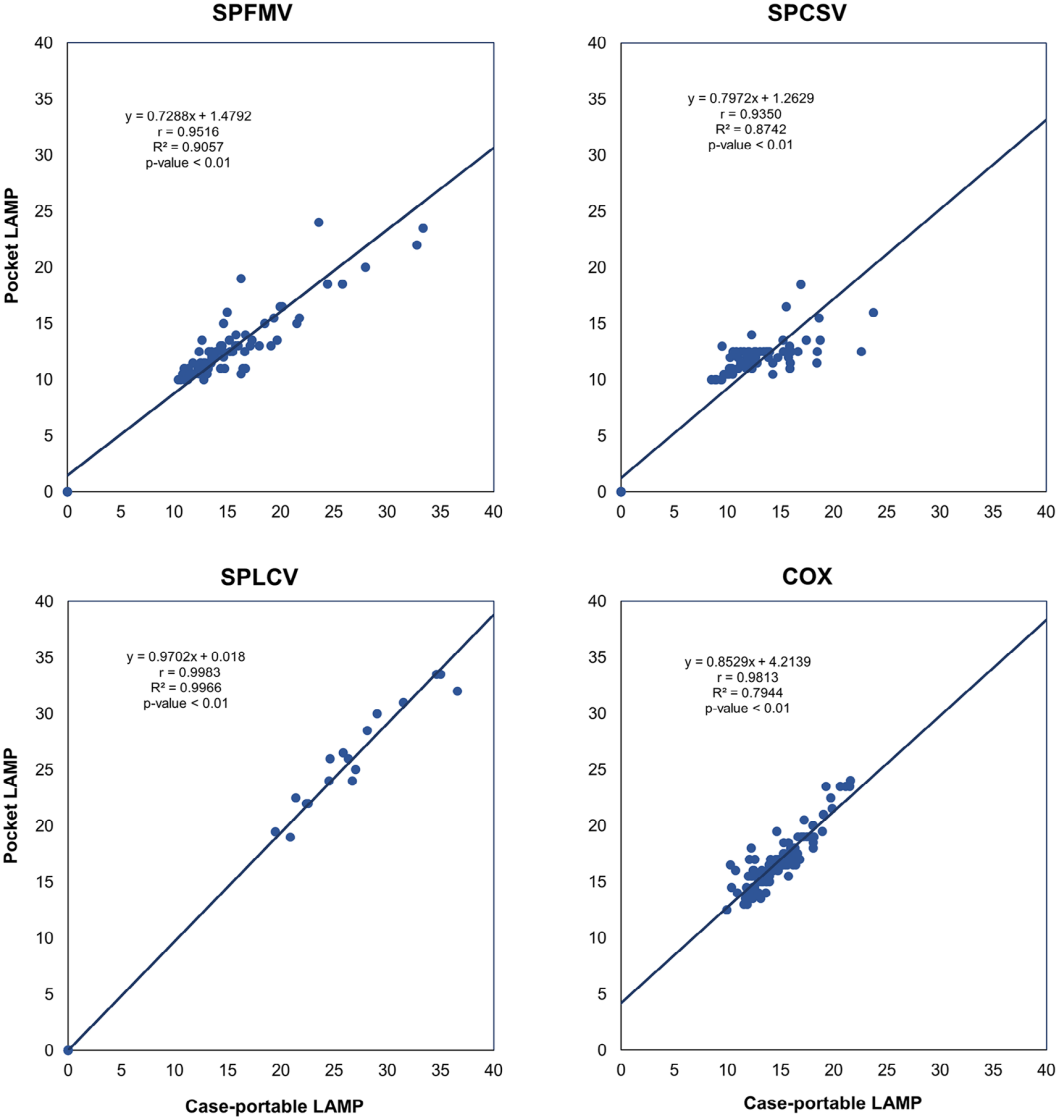
Correlation of Time to Positive (TTP) between two LAMP devices for sweetpotato virus detection. Each point represents an individual TTP value (in minutes) from a LAMP test. The correlation coefficient (r > 0.89) indicates a strong positive correlation between the devices. The coefficient of determination (R ^2^) shows that 79% to 99% of the variability in TTP from one device is explained by the other, indicating strong agreement. A p-value < 0.01 confirms the high statistical significant of the correlation.

Correlation analysis showed a strong relationship between TTP values from the pocket LAMP and case-portable devices ( Figure 5). A correlation coefficient (r > 0.89) indicates a very strong positive correlation between the measurements from both devices, with a p-value < 0.01 confirming a highly significant agreement.

The coefficient of determination (R ^2^) values, ranging from 0.79 to 0.99, indicate how closely the pocket LAMP device measurements match those of the case-portable one. This suggests that 79 to 99% of the variation in TTP measurements from the pocket LAMP device is related to those from the case-portable LAMP device ( Figure 5), indicating a strong agreement between measurements from the two devices. The unexplained variability (between 1% and 21%) could be related to detection thresholds, measurement noise, or device bias. Additionally, Deming regression further validated a strong linear association between the two devices for virus detection (Supplementary Figure S1, Fuentes et al., 2025).

### Cost-effectiveness analysis

The price of the case-portable device varies by supplier, so an average price was used for this analysis. There is a notable cost difference between implementing the two systems. On average, the case-portable device is priced at USD 15,890, whereas the total cost of implementing the pocket LAMP system, including accessories for eight simultaneous LAMP tests, is approximately USD 3,246. This makes the pocket LAMP system about 4.9 times more cost-effective ( Table 1). When considering local supplier pricing, the difference could be as high as 7 times.

**Table 1.** Cost comparison of pocket and case-portable LAMP devices for sweetpotato virus detection. Cost analysis includes required accessories and consumables, for eight simultaneous LAMP tests. The calculation is based on 2,000 individual tests, corresponding to 500 samples each analyzed for three sweetpotato viruses and an internal control.

Items	Pocket LAMP device (DoctorVida)		Case-portable LAMP device (Genie III)		Cost difference	
	Quantity	Cost (USD)	Quantity	Cost (USD)		
**Devices and accessories**					**4.9 times** (Devices and accessories)	**1.7 times** (Devices, accessories, and consumables)
Device (with carry case)	8	2,960	1	15,890 ^a^		
Smartphone	2	120 ^b^	0	0		
Internet (weeks)	20	44 ^b^	0	0		
Portable power bank (10,000mAh)	2	81	0	0		
USB 2.0 (4-Port) hub	2	22	0	0		
Multi-plugs	2	19	0	0		
Power adapters	8		0	0		
**Sub-total **		**3,246**		**15,890**		
**Cost influencing consumables**						
0.2 ml tubes, clear flat cap (Axygen PCR-02-C x 1,000)	2	140	0	0		
0.2 ml tubes (OptiGene OP-008-500 strips)	0	0	0.5	360		
Master mix, 300 tests (OptiGene ISO-DR004-RT) ^c^	14	8,748	7	4,374		
Hexadecane (Sigma-Aldrich H6703-100ml)	1	156	0	0		
**Sub-total **		**9,044**		**4,734**		
**Total**		**12,290**		**20,624**		

^a^ = Average price of case-portable ‘Genie III’ device, as prices vary by supplier.

^b^ = Costs calculated based on local prices in Tanzania.

^c^ = 50 μl for the pocket LAMP device and 25μl for case-portable device.

When including consumables, total expenses increase by USD 9,043 for the pocket LAMP system and USD 4,733 for the case-portable system. Consequently, the cost-effective difference is reduced to 1.7 times ( Table 1), though it could be as high as 2.2 times for local pricing.

## Discussion

The DoctorVida pocket LAMP device, initially designed for rapid detection in human health context, shows promising potential for plant pathogen diagnostics. In this study, we conducted 560 LAMP tests to evaluate its utility in detecting major sweetpotato viruses. Rather than comparing performance directly with the case-portable ‘Genie III’, currently the most widely used field-deployable LAMP platform, the study focused on assessing the suitability of this portable system under real-world conditions. The results demonstrated consistent performance across both systems, with high agreement observed for all target viruses. Statistical analyses, including correlation and regression modelling, confirmed a high degree of consistency, further validating the accuracy and reliability of the LAMP assays. Collectively, our results confirm the reliability of LAMP test method and support the utility of the pocket LAMP device as a viable field-deployable diagnostic tool for sweetpotato virus detection in seed production systems. Its portability and dependable performance present a promising opportunity to strengthen virus surveillance and improve seed health management.

To ensure a robust assessment of LAMP assay performance, we carefully selected samples that represented a wide range of symptom severity and potential virus loads. Additionally, the pocket LAMP device’s ability to operate with external power banks also enables field-based diagnostics. As long as there is internet connectivity, the results are saved and uploaded to the cloud (supplier’s server) for later data management. However, in remote locations with poor or no internet access, its use becomes more challenging. While results can still be viewed in real time on a cell phone screen, cloud storage and data synchronization may be delayed or unavailable.

The observation that SPLCV was detectable in fresh tissue but not in dried samples from the same samples highlights the importance of sample preservation methods. It seems that drying tissues may compromise some viruses’ detectability when using rapid plant sap extraction methods like Alkaline PEG buffer. Mark et al. (2024) evaluated the effect of sample storage time on the quality of DNA and RNA extracted from leaf tissues and concluded that the quality of nucleic acid degrades as time progresses, with significant degradation occurring over longer timeframes. That degradation may reduce the ability to detect a virus in the dried leaf samples.

We observed that the pocket LAMP device is sensitive to movement, improper cap closure, weak internet connection, and presence of small air bubbles in the reaction mixture inside the tube. Although any or all these things can affect the reliability of results, they are manageable in routine use.

The single-sample processing limitation of the pocket LAMP device could be addressed by operating multiple devices concurrently, depending on testing needs and available personnel. For instance, to run eight tests using the pocket LAMP setup would require eight devices and two smartphones, since one phone can control up to four devices.

Since initial tests in 2022, both the device and its associated mobile application have been enhanced for plant pathogen diagnostics, including expanded support for Android and iOS, improved reading channels, excitation LED light, and firmware updates for multiple incubations. Researchers can access information, create protocols, manage data through an online portal. Further improvements—such as integration of fluorescence derivative (-df/dt) graph analysis—may enhance its analytical capacity.

Cost analysis revealed that implementing pocket LAMP devices for LAMP assays is more economical, although the case-portable ‘Genie III’ offers other advanced features such as dissociation curves (which allow confirmation of specific amplification), storing results in the device without internet connection. The extent of cost savings depends on the comparison metrics: equipment, accessories, and consumables for the pocket LAMP setup cost approximately two times less, with per-test cost reduced by 40%, even though it requires more consumables—specifically hexadecane and double the master mix volume. The decision between these LAMP systems should be guided by operational requirements, desired throughput, and budget. It is important to consider whether the higher price of the case-portable ‘Genie III’ device is justified by its additional features or if the less expensive option is adequate for decentralized virus surveillance.

In conclusion, the pocket LAMP device offers a reliable, affordable, and adaptable diagnostic tool for sweetpotato virus detection, particularly suited for low-resource or decentralized contexts.

## Ethics and consent

Ethical approval and consent were not required.

## Reporting guidelines

Figshare: START checklist for ‘Evaluation of an ultra-portable pocket-sized device for running Loop mediated isothermal amplification (LAMP) assays for rapid detection of sweetpotato viruses’.

https://doi.org/10.6084/m9.figshare.29114084 ( Fuentes et al., 2025)

Data are available under the terms of the Creative Commons Attribution 4.0 International license (CC-BY 4.0).

## Data Availability

Figshare: Evaluation of an ultra-portable pocket-sized device for running Loop mediated isothermal amplification (LAMP) assays for rapid detection of sweetpotato viruses https://doi.org/10.6084/m9.figshare.29109401 ( Fuentes et al., 2025). The curated underlying data is available in Tables S3 and S4, provided in the extended data. Data are available under the terms of the Creative Commons Attribution 4.0 International license (CC-BY 4.0). Extended data referred to in the manuscript text as Supplementary materials are publicity available in: Figshare: Extended data for “Evaluation of an ultra-portable pocket-sized device for running Loop mediated isothermal amplification (LAMP) assays for rapid detection of sweetpotato viruses”. https://doi.org/10.6084/m9.figshare.29109401 ( Fuentes et al., 2025) Data are available under the terms of the Creative Commons Attribution 4.0 International license (CC-BY 4.0).
